# Recurrent Umbilical Pilonidal Sinus: An Uncommon Condition Successfully Treated With Omphalectomy and Umbilicoplasty

**DOI:** 10.7759/cureus.93714

**Published:** 2025-10-02

**Authors:** Lud Eyasu, Patrick D Melmer

**Affiliations:** 1 Surgery, Virginia Commonwealth University, Richmond, USA

**Keywords:** family medicine, general surgery consult, recurrent pilonidal sinus disease, umbilical pilonidal sinus, umbilicoplasty

## Abstract

Umbilical pilonidal sinus is a rare subtype of chronic pilonidal disease, which more commonly affects the sacrococcygeal region. Diagnosis is often delayed due to low clinical suspicion and overlapping presentations with more common abdominal wall conditions. We report the case of a 33-year-old male with a six-year history of intermittent malodorous umbilical drainage causing significant psychosocial distress. Previous conservative management, including antibiotics, hair removal, and hygiene measures, failed to provide adequate relief. Imaging, both ultrasound and computed tomography (CT), was unremarkable apart from only mild umbilical inflammation. Operative exploration revealed a nest of hair and sinus tracts terminating in a cyst at the umbilical base. Complete excision of devitalized tissue was performed, followed by umbilicoplasty, achieving a satisfactory cosmetic result based on patient-reported outcome. The patient recovered uneventfully with no recurrence at one year. Umbilical pilonidal sinus, though rare (~0.6% of pilonidal disease cases), should be considered in patients with chronic umbilical discharge. Surgical excision with umbilicoplasty offers definitive management and reliable long-term results.

## Introduction

Pilonidal disease is a chronic inflammatory condition most commonly affecting young men in the sacrococcygeal region, with an estimated global incidence of approximately 26 per 100,000 [1]. Umbilical involvement is extremely rare, accounting for approximately 0.6% of all pilonidal sinus cases [2]. Its pathogenesis involves penetration of hair and debris into the umbilical cleft, triggering chronic inflammation and sinus formation. Risk factors include male sex, young age, obesity, diabetes, deep umbilical clefts, poor hygiene, and hirsutism [3-4].

Because the clinical presentation of umbilical pilonidal sinus (UPS) often overlaps with more common conditions such as umbilical hernia, urachal cyst, sebaceous cyst, or omphalitis, diagnosis is frequently delayed. This can result in prolonged patient distress and repeated ineffective therapies. Previous literature has described both conservative measures (antibiotics, hair removal, hygiene improvement) and surgical strategies (simple excision, omphalectomy, and umbilical preservation techniques). Recurrence rates following surgery range between 5% and 12% in small series [5]. A recent randomized controlled trial comparing conservative versus surgical management demonstrated significantly better long-term outcomes with surgical excision [6].

Here, we present a case of recurrent UPS after failed conservative treatment, successfully managed with omphalectomy and umbilicoplasty. We highlight the diagnostic challenges, psychosocial burden, and importance of maintaining clinical suspicion even when imaging is inconclusive.

## Case presentation

A 33-year-old male presented with a six-year history of intermittent malodorous drainage from his umbilicus. He reported significant anxiety, difficulty maintaining employment, and relationship strain due to his symptoms. Multiple prior evaluations led to conservative management including antibiotics, hair removal, and strict hygiene, none of which provided lasting relief.

His medical history was notable for an overweight status with a BMI of 27. He denied smoking and had no history of diabetes, skin disease, or other systemic comorbidities. Physical examination was positive for a hirsute abdomen and umbilicus with mild erythema, tenderness, and a visible sinus opening with intermittent discharge. No palpable hernia, urachal mass, or fluctuance was detected.

**Figure 1:**
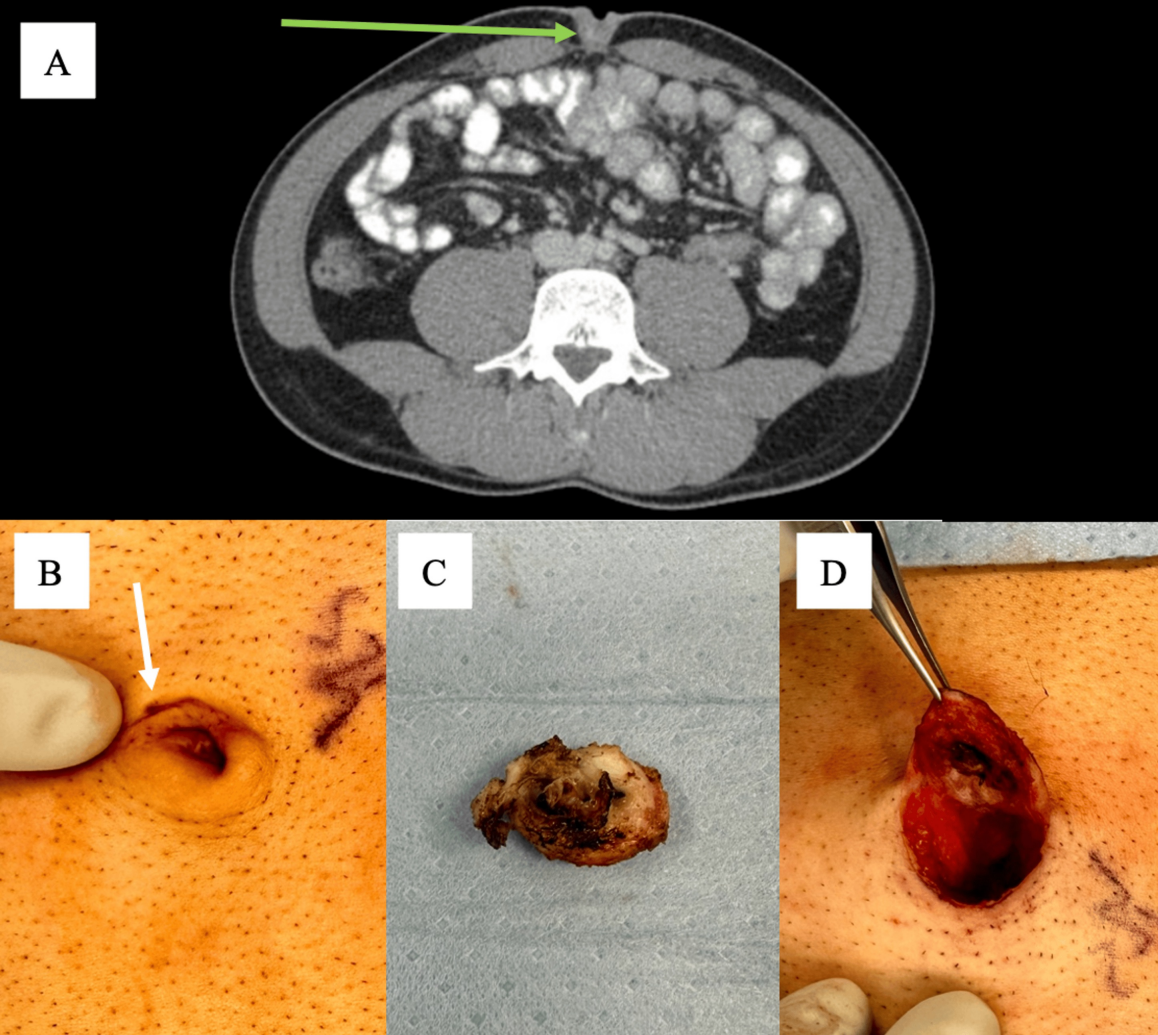
Umbilical pilonidal disease A: CT demonstrating umbilical inflammation (green arrow). B: Intraoperative photograph demonstrating pits (white arrow) surrounded by a large amount of shaved hair. C: Intraoperative photograph of resected umbilical pilonidal cyst, approximately 2.5cm in length. D: Intraoperative photograph of umbilicus following resection demonstrating resection cavity with overlying skin.

Prior ultrasound was negative, and CT demonstrated no evidence of urachal cyst, hernia, or mass and was positive only for mild umbilical inflammation (Figure 1A). Given the chronicity, failed conservative therapy, and clinical suspicion for UPS, operative exploration was recommended.

Intraoperatively, an extensive nest of hair and sinus tracts leading to a cyst at the umbilical base was identified (Figure 1B). Devitalized tissue and inflamed umbilical skin were completely excised (Figure 1C). An umbilicoplasty was performed for reconstruction, achieving a satisfactory cosmetic outcome (Figure 1D). The patient recovered uneventfully. At the one-year follow-up, he remained symptom-free and reported high satisfaction with the aesthetic outcome, though no standardized postoperative photograph was available.

## Discussion

UPS is an atypical clinical entity that presents diagnostic and therapeutic challenges. Its rarity, overlapping clinical features, and often inconclusive imaging contribute to delayed recognition. As in our case, ultrasound and CT may fail to demonstrate sinus tracts or hair nests, underscoring the importance of clinical suspicion. When symptoms persist, surgical exploration should be considered despite negative imaging.

Risk factors for UPS include hirsutism, obesity, deep umbilical cleft, local moisture, and poor hygiene [2-4]. Our patient’s hirsute abdomen and overweight status likely contributed to recurrent hair impaction and inflammation. Conservative measures alone are generally ineffective, especially in recurrent disease. In this case, multiple attempts at conservative therapy failed over six years.

Surgical excision remains the gold standard for definitive treatment. Reported approaches include simple sinus excision with umbilical preservation, complete omphalectomy, and reconstruction with umbilicoplasty [4]. Recurrence rates after surgery are relatively low (5-12%), but complete excision and attention to predisposing factors are critical to success [5].

Our case contributes to the literature by emphasizing several points. First, UPS may present with years of symptoms despite negative imaging, emphasizing the need for high clinical suspicion. Second, psychosocial distress is a significant and underreported impact of UPS, which can be addressed and improved with definitive operation. Third, recurrent or refractory cases benefit from surgical excision with umbilicoplasty and can achieve high levels of patient satisfaction.

## Conclusions

UPS, though rare, should be included in the differential diagnosis of chronic umbilical discharge. Clinicians must maintain a high index of suspicion, particularly in patients with risk factors such as hirsutism and obesity, even when imaging is unrevealing. This case underscores the importance of considering UPS early to avoid years of ineffective therapy and psychosocial burden. Definitive surgical excision with umbilicoplasty provides durable symptom resolution and satisfactory cosmetic outcomes.
